# Genomic Characterization of the Istrian Shorthaired Hound

**DOI:** 10.3390/ani10112013

**Published:** 2020-11-01

**Authors:** Ivona Djurkin Kušec, Ivica Bošković, Minja Zorc, Kristina Gvozdanović, Dubravko Škorput, Peter Dovč, Goran Kušec

**Affiliations:** 1 Faculty of Agrobiotechnical Sciences Osijek, Josip Juraj Strossmayer University of Osijek, Vladimira Preloga 1, 31000 Osijek, Croatia; idurkin@fazos.hr (I.D.K.); ivica.boskovic@fazos.hr (I.B.); kristina.gvozdanovic@fazos.hr (K.G.); goran.kusec@fazos.hr (G.K.); 2 Department of Animal Science, Biotechnical Faculty, University of Ljubljana, Jamnikarjeva 101, 1000 Ljubljana, Slovenia; peter.dovc@bf.uni-lj.si; 3 Faculty of Agriculture, University of Zagreb, Svetošimunska cesta 25, 10000 Zagreb, Croatia; dskorput@agr.hr

**Keywords:** Istrian shorthaired hound, genetic diversity, population structure, genealogical data, SNP array

## Abstract

**Simple Summary:**

Istrian shorthaired hound is an old indigenous Croatian dog breed. However, there is no data available about its genetic diversity, population structure, and inbreeding level, which would be needed for advanced management and conservation of this breed. We studied the population structure using 220K SNP array and compared the genomic data with the genealogical records. This allowed us to place Istrian shorthaired hound on the map of the world dog populations. The Istrian shorthaired hound has a relatively high effective population size and low level of inbreeding. Its genome contains a higher number of short runs of homozygosity compared to other analysed breeds, thus confirming the old origin of the breed and balanced use of breeding animals. The breed was placed in the same clade with Italian hunting breeds suggesting that the Istrian shorthaired hound was probably used for the development of some of these breeds. The genomic analysis importantly contributes to the development of future breeding strategies and supports the conservation of the Istrian shorthaired hound.

**Abstract:**

Istrian shorthaired hound is an old indigenous Croatian dog breed with historical traces of its origin, which date back to the 14th century. Due to its intelligence and great hunting abilities, it is considered an excellent hunting dog. Despite its ancient origin, there is no data on genetic diversity, population structure, and degree of inbreeding that could be used for advanced management and conservation of this breed. Our study aimed to provide a high-resolution population structure of the Istrian shorthaired hound using a 220K HD SNP array, to compare the obtained data with the genealogical records and to place the breed in a broader context of world dog populations. Relatively high population size and low inbreeding coefficient estimated from genealogical data indicate a preserved genetic diversity in this breed. The principle component analysis, the NeighborNet network, and TreeMix were used to determine the genetic relationship between the Istrian shorthaired hound and other breeds. The Istrian shorthaired hound was found to be genetically related to Italian hunting dogs sharing the same branch with the Segugio Italiano a Pelo Raso and Segugio Italiano a Pelo Forte. The ADMIXTURE analysis indicated that the Istrian shorthaired hound could be involved in the development of some other hunting dog breeds. The estimated effective population size (Ne) based on SNP data was similar to Ne calculated from genealogical data indicating the absence of bottlenecks and well-balanced use of breeding animals. The low genomic inbreeding coefficient, together with the higher number of short runs of homozygosity, observed in the Istrian shorthaired hound, confirms the ancient origin of the breed based on historical documents. The analysis of selective sweeps identified genomic regions with the strongest selection signals in the vicinity of the genes associated with cognitive performance and behavior. Genome analysis proved to be a useful tool for estimating population parameters and can be implemented in the conservation plan for this indigenous breed.

## 1. Introduction

**Figure 1:**
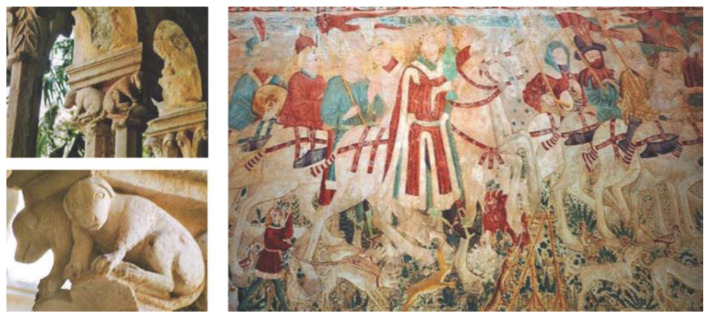
Historical traces of the breed’s origin [2].

Istrian shorthaired hound is an old Croatian indigenous dog breed believed to descend from the old type of the “East Adriatic white hound with markings”. The first evidence of its origin was found in the Franciscan monastery in Dubrovnik, which was built between 1327 and 1328 A.D., while the first painted evidence can be seen on the painting “The bow of the three kings” in the cemetery chapel next to the village of Beram in Istria from 1474 (Figure 1). The first written proof of the existence of the breed dates back to 1719, in the manuscript “*De vita Populi et de Cultura armentorum et pecorum Diacovae et eius Districtus anno Domini 1719*” (“On the living population and cattle breeding in Đakovo and surroundings in 1719”), where the Bishop of Đakovo, Petar Bakić, described a dog “three to four feet tall, with short or medium-length white hair and red markings, and which is raised mainly in the coastal areas of Croatia”. This description corresponds well to the appearance of the Istrian shorthaired hound. In the medieval period, Istria was a part of the Venetian Republic, but the region of Pazin and the east coast of Istria belonged to Charles the Sixth of the Habsburgs. That is the reason why breeding of this type of dog was known and also practised in geographically separated parts of Croatia. In the above-mentioned manuscript, the Bishop Bakić also reported that in the 14th century there were several hunting dog breeds of high quality, which were frequently exported to other countries for the improvement of their local populations of hunting dogs [1].

**Figure 2:**
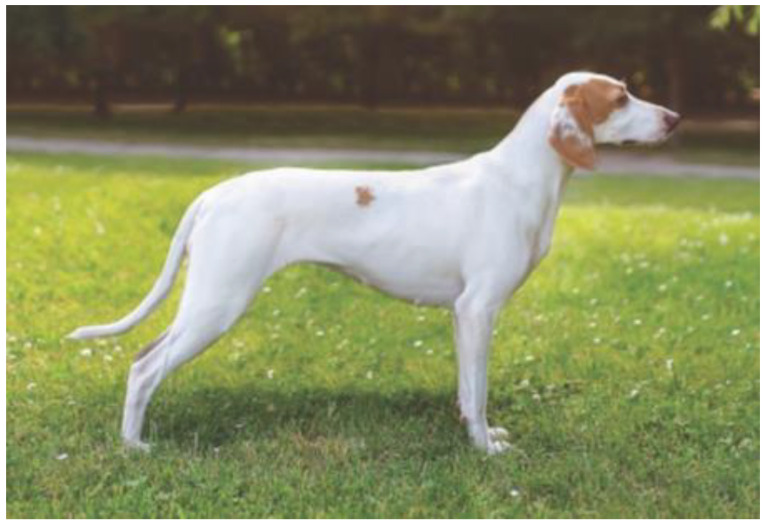
Istrian shorthaired hound [2].

The breed was recognized by the F.C.I. (Fédération Cynologique Internationale) in 1955 when its first standard was published. According to the standard, it is a dog of medium size, classified in Group 6 of the F.C.I. breed nomenclature (“Scent hounds and related breeds”). The body of the animal should be hound-like strong, yet elegant with harmonious movements. The hair should be short, fine, and snow-white, with orange markings on the head and body (Figure 2).

Istrian shorthaired hound has a very gentle but determined temperament, and the breed represents one of the most appreciated scent dogs among hunters due to its extraordinary endurance, precision, and speed, with a clear and strong voice during a pursuit of the game (Fédération Cynologique Internationale (FCI), Breed standard no. 151, 2015). However, despite its ancient origin and well-known qualities, the number of puppies descending from this breed is constantly decreasing [3], with a total of 862 Istrian shorthaired hound puppies born in 2019, as registered by the Croatian Kennel Club.

Until today there is no official cynotechnical management programme for the breed, i.e., the population structure depends solely on the decisions of individual breeders. This can potentially lead to a high incidence of the “popular sire effect” [4] and loss of genetic diversity, especially in small populations such as the Istrian shorthaired hound. Except for the study of Zajc and Kus (2001) [5] on the Slovenian population of Istrian shorthaired hound, more comprehensive genetic studies have not been reported for this breed. The abovementioned study was performed using an early set of microsatellite markers [6] having lower informativeness [7] and reflecting changes in a shorter time interval than SNPs [8].

Our study aimed to provide a high-density genomic representation of the population structure and to compare the obtained genomic data with the genealogical data from pedigree records. Also, we studied the relationship of the Istrian shorthaired hound with other dog breeds to place the breed in a broader context of world dog populations and to study its relationship with other hound dogs and related breeds originating from the same geographical region. Our results provide a solid basis for an advanced selection program that could more effectively monitor genetic diversity and prevent future bottlenecks in this breed.

## 2. Materials and Methods

### 2.1. Sample Collection and DNA Extraction

Buccal samples of 48 adult dogs were collected on dog exhibitions and hunting competitions organised by the Croatian Kennel Society and Croatian Hunting Society, after getting consent from dog owners. Animals selected for the DNA analysis were as distantly related as possible according to their pedigree information (no full siblings were included in the analysis). Buccal cell DNA was extracted using the Sbeadex livestock kit (LGC Genomics GmbH, Berlin, Germany) according to the manufacturer’s protocol. The concentration and the purity of the genomic DNA were assessed using the NP80 NanoPhotometer (Implen GmbH, Munich, Germany).

### 2.2. SNP Genotyping, Assembly of the Merged Dataset, and Quality Control

Samples were genotyped using Illumina CanineHD BeadChip (Illumina Inc., San Diego, CA, USA) containing 220,853 SNPs. The obtained dataset was merged with the publicly available SNP chip data downloaded from NCBI Gene Expression Omnibus (GEO) and Dryad (Table 1). Illumina GenomeStudio software (v2011.1) was employed to perform primary data analysis, including raw data normalisation, clustering, and genotype calling for data obtained from the GEO. Four publicly available datasets were merged with our data, and only breeds with five or more samples were included in the analysis. If the breed contained more than 48 samples, a subset of 48 samples per breed was selected randomly. SNPs on X and Y chromosomes, SNPs with a call rate <95%, and samples with a call rate <90%, were removed from the data set using SNP and Variation Suite v8.9.0 (Golden Helix, Inc., Bozeman, MT, USA, http://www.goldenhelix.com). The final dataset contained 3093 animals from 151 dog breeds, 7 Apennine wolves, and 14 Grey wolves with 144,168 common SNPs. Accession numbers of all samples included in the study are provided (Supplementary Table S1). The genotype data generated in the present study are available from the Dryad Repository (doi:10.5061/dryad.1vhhmgqrj).

### 2.3. Genealogical Analysis

The genealogical analysis was performed on the pedigree records from 48 individuals selected for genomic analysis, spanning four generations. The database records for each dog contained its identification number, sex, date of birth, as well as identification numbers and date of birth of its sire and dam, their parents and grandparents. The basic pedigree structure was detected using CFC software [12]. The total pedigree file contained 520 individuals. The pedigree quality and integrity were described by the average number of maximum generations traced back, the average number of full generations, and the average number of complete equivalent generations. The average relatedness coefficient of each individual was calculated, according to Gutierrez et al. (2003) [13]. The inbreeding coefficient of an individual was computed by the algorithm implemented in ENDOG [14]. Effective population size (Ne) was calculated as Ne = 1/(2 ΔF) [15] using the same software.

### 2.4. Genetic Parameters

Populations module of Stacks v2.5.2 software [16] was used to compute the average frequency of most common allele, observed heterozygosity (H_OBS_), expected heterozygosity (H_EXP_), nucleotide diversity (π), and inbreeding coefficient (F_IS_). Effective population size (Ne) was estimated from linkage disequilibrium (LD) in the SNeP software [17].

### 2.5. Runs of Homozygosity

SNP and Variation Suite v8.9.0 was used for the identification of runs of homozygosity (ROH) in each individual. ROH were defined as runs of 25 or more homozygous SNPs that had a minimum run length of 500 kb. No heterozygous SNPs and not more than five missing SNPs were allowed. ROH were summarized in three length categories: short (ranging from 0.5 to 2.5 Mb); medium (ranging from 2.5 to 5.0 Mb); and long (longer than 5 Mb). The average sum of ROH per breed was visualized as a bar plot using the library for R ggplot2 [18]. Genomic inbreeding coefficients (F_ROH_) were calculated as described by McQuillan et al. (2008) [19]. The length of the autosomal genome was set to 2,392,715,236 bp. ROH islands, regions where more than 70% of samples shared ROH, were identified. The incidence of markers in ROH was determined and visualized as a Manhattan plot using SNP and Variation Suite v8.9.0 Genomic regions harbouring ROH islands were analysed using BioMart and Ensembl, release 101.

### 2.6. Genetic Relationships and Population Structure

#### 2.6.1. Genomic Relationships and Genetic Distances

Principal component analysis (PCA) was performed using SNP and Variation Suite v8.9.0 and visualised using Scatterplot3D function in JMP^®^, Version 9.0. SAS Institute Inc., Cary, NC, USA, 1989–2019. PCA-scatter plot (i.e., first three PCs) was used to highlight a subset of dog breeds for further analyses. The subset selected for further analyses was visualized as 2D PCA using ggplot2 package [18]. Pairwise Nei’s genetic distances [20] between breeds were calculated using the StAMPP package [21] for R. The NeighborNet network was constructed and displayed using SplitsTree5 software [22].

#### 2.6.2. Population Structure

Relationships between breeds and population structure were analyzed for a subset of 31 dog breeds (Supplementary Table S1) and a Grey wolf. Dog breeds were selected according to their geographic location, type of the breed, and possible historical connections among them. The SNP and Variation Suite v8.9.0 was used for LD pruning. Pairs of markers within 50 bp window moving five bp were compared with each other to measure their pairwise LD (r2) using the composite haplotype method (CHM). If a pair of markers within the window was in LD greater than 0.5, the first marker in the pair was pruned. In total, 43,953 markers were pruned in the dataset.

Population structure for a set of 31 dog breeds and Grey wolf was evaluated using a model-based clustering algorithm Admixture 1.3.0 [23]. To avoid sample size related bias, the dataset was resized to a maximum of 15 randomly selected individuals per breed. The program was run from K = 2 to K = 40. The optimal number of clusters was estimated through 20-fold cross-validation of the accuracy to assign samples to the correct cluster. Clumpak software [24] was used for the visualization of results. PLINK v1.90 [25] was used for removing sites with missing data and for the conversion of data to the format appropriate for TreeMix analysis. After removing sites with missing data, 44,352 markers remained. TreeMix software [26] was used for modelling the relationships and possible gene flow among the Istrian shorthaired hound, a subset of 14 dog breeds, and Grey wolf as a root. Trees with 1–8 migration events were explored.

### 2.7. Genome-Wide Scan of Selection Signatures

Data were phased with BEAGLE V4 software [27] using default settings. Signature of selection analysis was performed using phased SNP data. The rehh package [28] in R [29] was used to detect within-population signatures of selection using the integrated haplotype score (iHS) [30]. The putative selective sweeps detected by iHS were annotated using Ensembl, release 101, and visualized as Manhattan plot using the *manhattanplot* function provided by rehh package.

## 3. Results

### 3.1. Genealogical Data

Population parameters obtained from genealogical records are presented in Table 2. The entire data set consisted of 520 animals; 54 of them were inbred (F > 0.00). The average inbreeding coefficient calculated from genealogical data was 0.0042 (0.42%), indicating a balanced use of breeding animals in the population and well-preserved genetic variability.

### 3.2. Population Genetics Statistics

Population genetics statistics for the Istrian shorthaired hound, 31 dog breeds, and Grey wolf were calculated and presented in Table 3. According to the percentage of polymorphic loci, Great Pyrenees (94.382), Istrian shorthaired hound (93.994), and Ibizan hound (93.536) represent a group of breeds with more than 93.5% of polymorphic loci. The observed (H_OBS_) and expected heterozygosity (H_EXP_) ranged from 0.185 (Lupo Italiano) to 0.339 (Pastore d’Oropa) and from 0.174 (Lupo Italiano) to 0.336 (Ibizan Hound), respectively (Table 3). The average H_OBS_ (0.317) and H_EXP_ (0.311) in the Istrian shorthaired hound were similar to values determined for Segugio Italiano a Pelo Forte, Segugio Italiano a Pelo Raso, Spinone Italiano, and Pastore della Lessinia e del Lagorai. The average nucleotide diversity (π) for the Istrian shorthaired hound was relatively high (0.314), and Istrian shorthaired hound had together with five other dog breeds (Bracco Italiano, Lupo Italiano, Otterhound, Pastore d’Oropa, Pastore della Sila) a negative inbreeding coefficient (Fis = −0.006), indicating an excess of heterozygosity. Low inbreeding coefficient (F_PED_) in Istrian shorthaired hound was also obtained from genealogical data (F_PED_ = 0.42%). The effective population size estimated from SNPs for the Istrian shorthaired hound was 109, similar to Ne obtained from genealogical data (111.24), confirming the accuracy of pedigree data. The population statistics for 32 dog breeds and Grey wolf are summarised in Table 3.

### 3.3. Runs of Homozygosity

**Figure 3:**
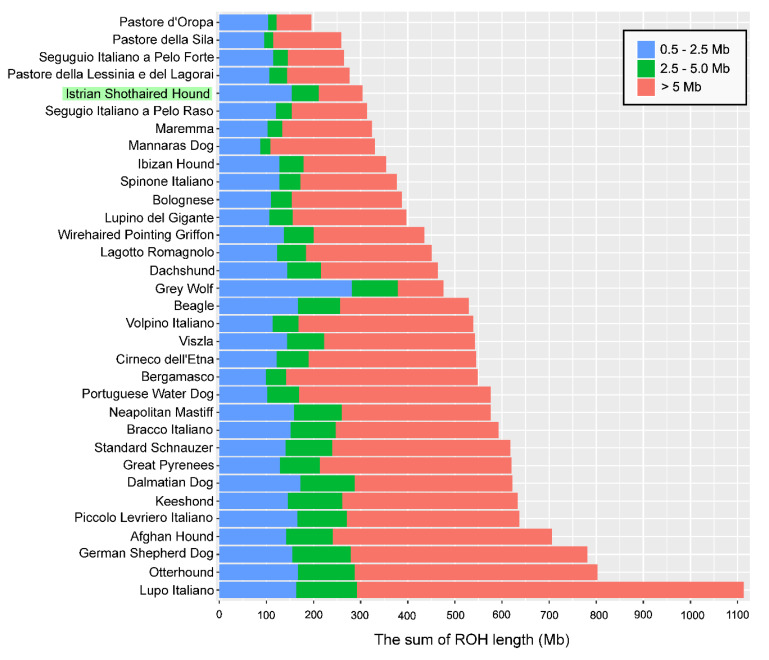
Average sum of ROH for the Istrian shorthaired hound, Grey wolf, and other 31 representative dog breeds. Blue: short (0.5 Mb ≤ ROH < 2.5 Mb), green: medium (2.5 Mb ≤ ROH < 5 Mb), and red: long (ROH > 5 Mb) ROH per breed.

Runs of homozygosity (ROH) were identified for each animal sample in the dataset. Additionally, cumulative and average ROH length, as well as the proportion of the genome that is located within the ROH (F_ROH_) were assessed. Figure 3 presents bar plots of ROH lengths for selected dog breeds grouped into three categories according to their size. Short ROH reflect LD (linkage disequilibrium) patterns, medium-sized are related to genetic drift, while the longest ROH originate from a recent ancestor [31]. The bar plot shows that the Istrian shorthaired hound has a significant number of short ROH and a small number of medium-sized and long length ROH compared to other dog breeds.

The frequency of ROH and their length-distribution differed across the Istrian shorthaired hound and four dog breeds that clustered together and are in close relation with the Istrian shorthaired hound either according to genomic data or because of their geographical origin (Table 4). The mean sum of ROH segment coverage was higher for short ROH than for long ROH in all five analyzed breeds.

Compared to other dog breeds, the Istrian shorthaired hound had the lowest mean length of ROH and the shortest lengths of long ROH segments. Furthermore, the Istrian shorthaired hound had the lowest mean ROH coverage; approximately 52% of the genome length covered by ROH was within the short ROH category (0.5–2.5 Mb). Accordingly, in Istrian shorthaired hound long ROH covered around 31% of the ROH covered genome, which was the lowest proportion in all investigated breeds.

To identify recent and ancient inbreeding, F_ROH_ was calculated for different ROH length classes. The mean genome inbreeding coefficient (F_ROH_) ranged between 0.25 for Bracco Italiano to 0.11 for Segugio Italiano a Pelo Forte. Compared to the other four dog breeds, the Istrian shorthaired hound exhibited the lowest F_ROH_ for long ROH segments (0.04), indicating that recent inbreeding is negligible in this dog breed.

### 3.4. Genetic Relationships and Population Structure

**Figure 4:**
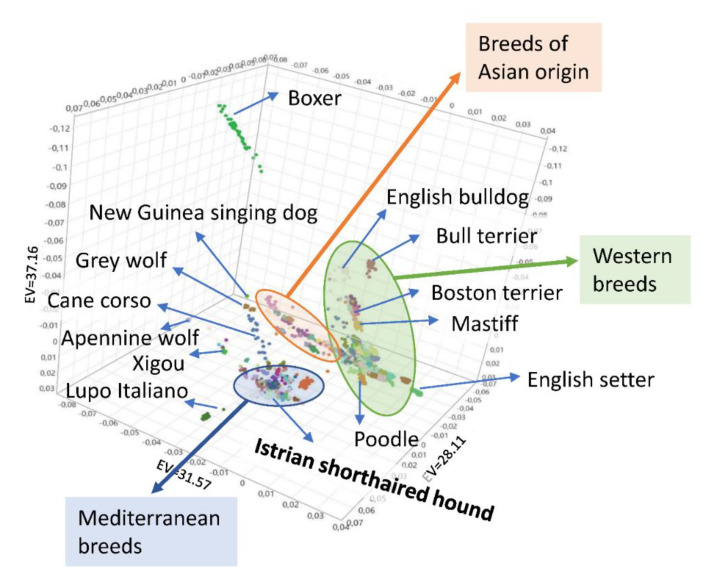
PCA-scatter plot of 151 dog breeds and two wolf populations.

**Figure 5:**
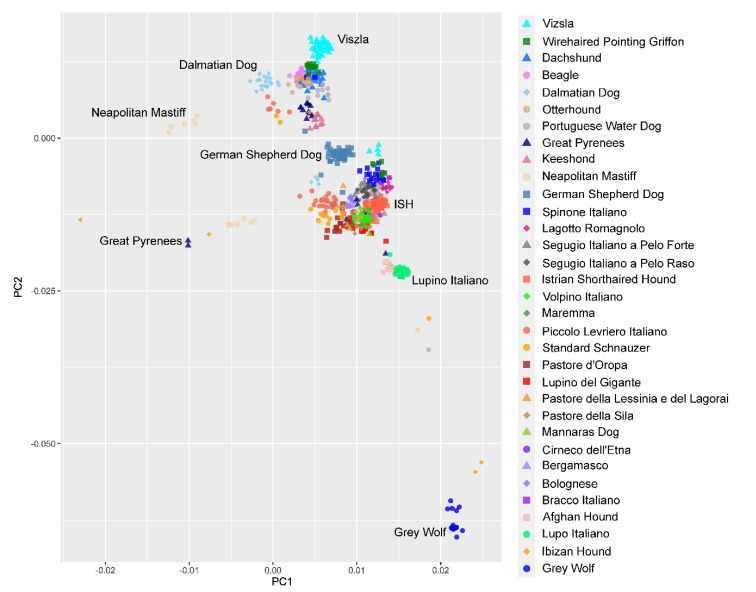
Zoom-in of the Mediterranean breeds cluster (Figure 4), highlighting a subset of 665 dogs which belong to 31 breeds related to the Istrian shorthaired hound (ISH).

The relationship of Istrian shorthaired hound and other dog breeds was visualized using principal component analysis (PCA) and NeighborNet network. PCA illustrates the population stratification of 3076 dogs from 151 breeds, seven Apennine-, and 14 Grey wolves. In the PCA analysis, the dog breeds formed three large groups corresponding to their geographical origin, representing breeds of Western, Asian, and Mediterranean origin (Figure 4). The Istrian shorthaired hound appeared to be genetically distant from all other breeds included in our study and was situated in the group of Mediterranean breeds, together with the majority of Italian hunting dog breeds (Figure 5). Together, they build the central part of the compact Mediterranean cluster of the breeds.

**Figure 6:**
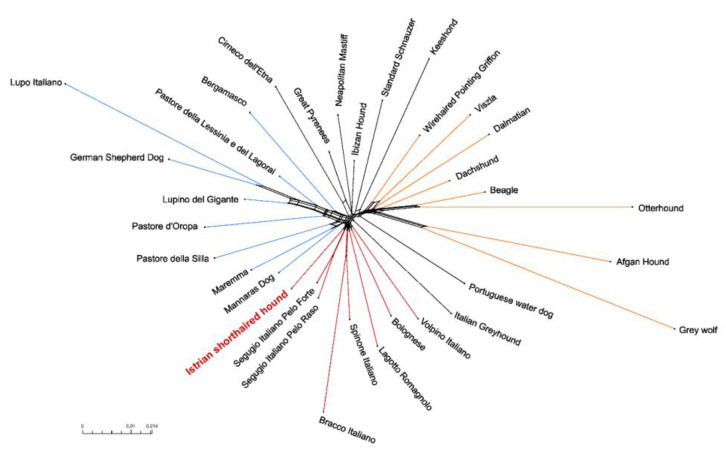
NeighborNet network computed from pairwise Nei distances between Istrian shorthaired hound, 31 other dog breeds, and Grey wolf.

The NeighborNet network was constructed to show the relationship between the Istrian shorthaired hound and Italian breeds, together with other dog breeds as representatives of the Western breeds and breeds of Asian origin (Figure 6). The NeighborNet network of selected dog breeds showed a close relationship of the Istrian shorthaired hound with Segugio Italiano a Pelo Forte, Segugio Italiano a Pelo Raso, Spinone Italiano, Bracco Italiano, Lagotto Romagnole, Bolognese, and Volpino Italiano. Istrian shorthaired hound and both Segugio Italiano breeds (a Pelo Forte and a Pelo Raso) shared the same branch of the clade, which indicates their genetic relatedness.

**Figure 7:**
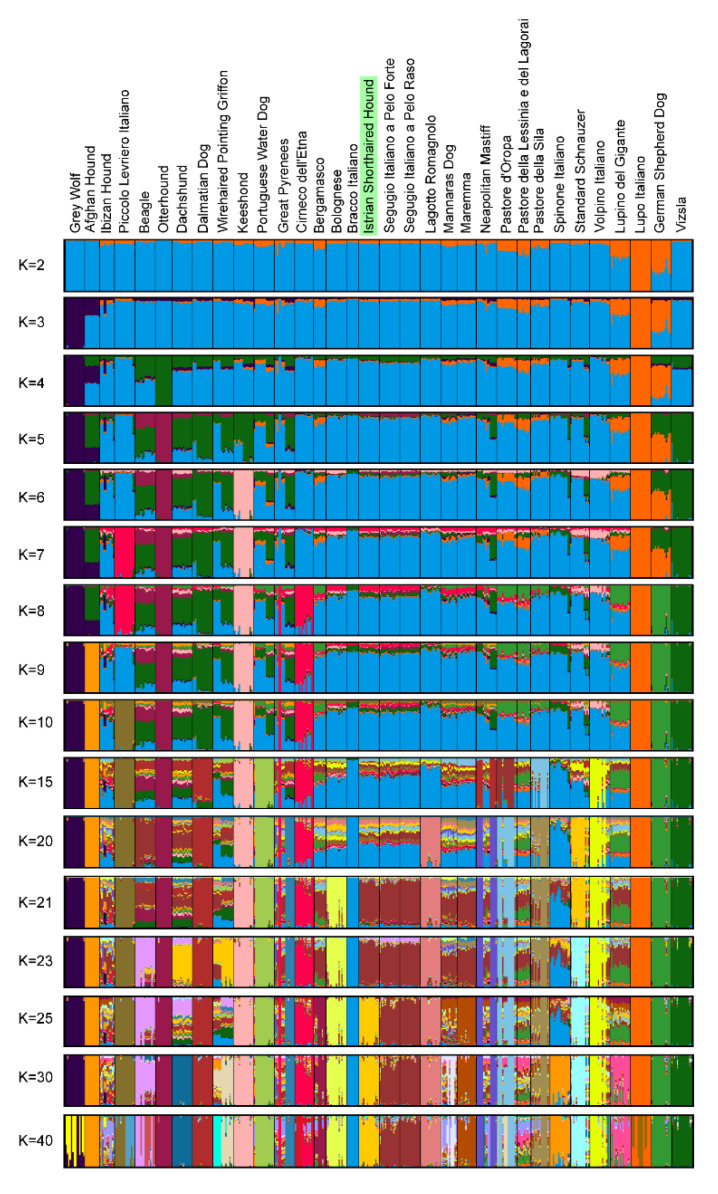
Population structure of Istrian shorthaired hound, 31 other dog breeds, and Grey wolf. Bayesian clustering performed with ADMIXTURE assuming K = 2 to K = 40. Each cluster is presented with a different color, and each individual is shown as a vertical bar representing the proportion of membership to a certain population.

**Figure 8:**
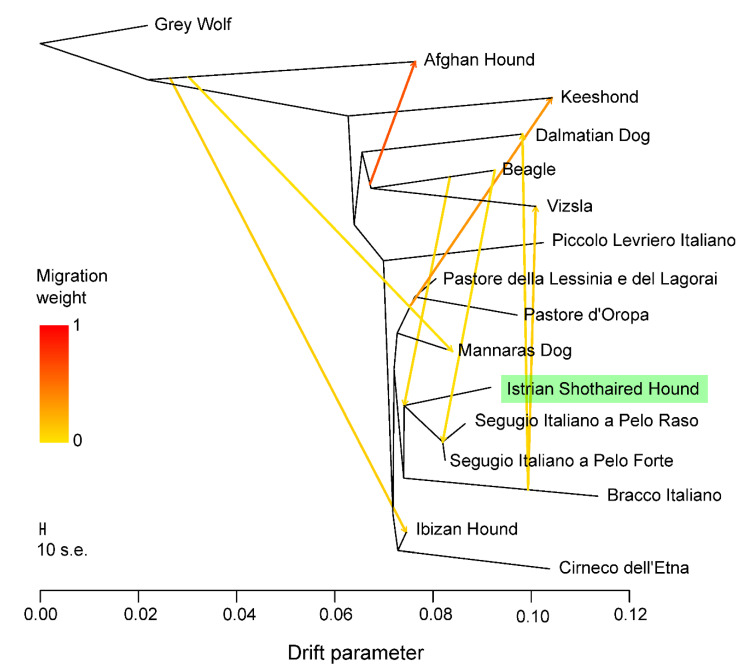
Graph inferred by TreeMix for the Istrian shorthaired hound, 14 dog breeds, and Grey wolf as root, allowing eight migration events. Migration arrows are colored according to their weight. Horizontal branch lengths are proportional to the amount of genetic drift that occurred on the branch.

The ADMIXTURE analysis revealed population structure and relationships between Istrian shorthaired hound breed, 31 other dog breeds, and Grey wolf (Figure 7). The optimal number of estimated clusters through 20-fold cross-validation was K = 30 (Supplementary Figure S1) when Istrian shorthaired hound separates from the two most related Italian breeds, Segugio Italiano a Pelo Forte and Segugio Italiano a Pelo Raso. In our analysis, Gray Wolf, Otterhound, Lupo Italiano, and German Shepherd Dog separated first from the Meditaranean cluster of breeds (K = 4), followed by Afghan Hound, Beagle, Dachshound, Dalmatian, Wirehired Poinitg Griffon, Keeshound, and Viszla (K = 5). With the exemption of Cirneco dell’Etna, which separates at K = 8, the central cluster of Mediterranean breeds remains relatively stable, with several breeds in the cluster, including Istrian shorthaired hound, showing moderate traces of admixture. At K = 23 the contribution of Beagle to Istrian shorthaired hound, Segugio Italiano a Pelo Forte, and Segugio Italiano a Pelo Raso can be detected, which is in line with the result of the TreeMix analysis, where migrations of Beagle to the ancestors of these three breeds were indicated (Figure 8). At K = 21 the group of three breeds (Istrian shorthaired hound, Segugio Italiano a Pelo Forte, and Segugio Italiano a Pelo Raso) separates from the central cluster and at K = 25 Istrian shorthaired hound separates from the other two closely related breeds.

In the TreeMix tree (Figure 8), Istrian shorthaired hound stems from the same branchpoint with ancestors of Segugio Italiano a Pelo Raso and Segugio Italiano a Pelo Forte. We only detected a weak genetic exchange between the ancestors of Beagle and the ancestors of the modern Istrian shorthaired hound. However, the migration edge from Beagle to the ancestor of Segugio breeds might reflect the relatively recent contribution of Beagle to Segugio Italiano a Pelo Raso and Segugio Italiano a Pelo Forte.

### 3.5. Genomic Patterns of Homozygosity

**Figure 9:**
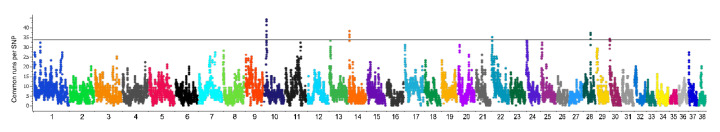
Manhattan plot of the distribution of ROH islands in 48 Istrian shorthaired hound genomes. The *y*-axis shows the number of overlapping ROH shared among individuals for each SNP. ROH islands, genomic regions where 70% or more samples shared ROH, were identified on chromosomes 10, 14, 22, 28, and 30.

Genomic regions with reduced diversity in Istrian shorthaired hound were identified on chromosomes 1, 10, 14, 22, 23, 24, 28, and 30 (Figure 9).

Our analysis was focused on the regions with the highest frequencies of ROH present in more than 70% of individuals, so-called ROH islands (Table 5). In Istrian shorthaired hound, ROH islands harbor genomic regions, which have already been associated with dog traits such as ear morphology, sport-hunting dog phenotype, and pointing phenotype (Table 5).

### 3.6. Genome-Wide Scan of Genome Signatures

Istrian shorthaired hound is considered as one of the best hare dogs among hunters. They are extremely good at detecting hare and are famous for their persistence in hunting (Fédération Cynologique Internationale (FCI), Breed standard no. 151, 2015). The analysis of selective sweeps using iHS statistics identified several genomic regions, with most of the SNPs with the strongest signals being associated with genes affecting the cognitive performance and behavior (Table 6).

**Figure 10:**
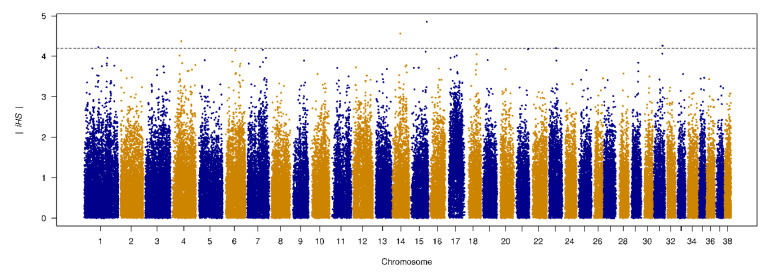
Manhattan plot of genomic selection regions detected using iHS. The strongest signals were detected on chromosomes 15, 14, 4, 31, and 1.

The SNP with the strongest iHS signal on CFA1 (Figure 10) is in the proximity of the phosphodiesterase 10A (*PDE10A*) gene. Strong signals were also found in *DGKB* (diacylglycerol kinase, beta), *RGS14*, and in the proximity of the *LMAN2* gene.

## 4. Discussion

### 4.1. Population Parameters, Inbreeding, and Effective Population Size

The average inbreeding coefficient (F) in Istrian shorthaired hound computed from genealogical data was relatively low (0.42%), and it was one of the lowest reported for hunting dog breeds in general (Table 7). The average F in other hunting dog populations ranged from 1.1% in the Italian population of Basset hound [37] to 33% in Norwegian Lundehund [38]. However, the majority of dog breeds has moderate average inbreeding coefficients.

The negative effects of inbreeding are usually the result of the rate at which inbreeding increases over time [46]. For this reason, population inbreeding is usually measured by the inbreeding rate per generation (ΔF). In the analyzed population of the Istrian shorthaired hound, the inbreeding rate ΔF was 0.45% with the mean equivalent generation of 1.70. In the Bavarian mountain hound, Hanoverian hound, and Tyrolean hound, the calculated ΔFs were 0.69%, 0.98%, and 1.88%, respectively [41]. In the Norwegian Lundehound, the estimated average inbreeding rate per generation was 4% [38]. The high ΔF observed in this Norwegian breed is the result of a very small effective population size. On the other hand, the estimated effective population size in the Istrian shorthaired hound was 111.24, which indicates a favourable genetic diversity of this breed and agrees with the low average inbreeding coefficient. According to Boichard et al. (1997) [47], the ratio between the effective number of founders and the effective number of ancestors shows whether the population passed a bottleneck. The ratio between these two parameters in the Istrian shorthaired hound was 0.59, which indicates the absence of detectable bottlenecks in this breed.

The analysis of SNP array data revealed the average nucleotide diversity of 0.314 in the Istrian shorthaired hound, which places Istrian shorthaired hound to the upper 30% of analyzed breeds, according to this parameter. Nucleotide diversity is the measure of genetic heterogeneity within the population and is analogous to genetic diversity [48]. The observed heterozygosity in Istrian shorthaired hound was 0.317, and it was the fifth most heterozygous breed in our dataset. In the study based on 16 microsatellite markers [5], even higher average heterozygosity (0.485) in the Istrian shorthaired hound population was reported. However, this difference is probably due to a different marker system used (microsatellites vs. SNPs) and to some extent also to the decreasing population size of the breed in the last two decades [3]. Istrian shorthaired hound together with five other dog breeds (Bracco Italiano, Lupo Italiano, Otterhound, Pastore d’Oropa, Pastore della Sila) had a negative inbreeding coefficient (Fis = −0.006), indicating an excess of heterozygosity [49] and a low rate of related animals, which might at least partly also be a consequence of our sampling strategy.

In the genomic context, the inbreeding coefficient is measured by F_ROH_, defined as a proportion of the autosomal genome in which autozygosity is derived from the assumption that very long stretches of homozygosity can only result from inbreeding [50]. This parameter proved to be very useful not only in tracking the population history, especially in consanguinity but also in the calculation of inbreeding depression and identification of lethal and semilethal genes segregating in a certain population. The inbreeding coefficient determined using ROH in Istrian shorthaired hound was 0.123, which is the median value among analyzed dog breeds (Table 3). The Istrian shorthaired hound had the lowest mean ROH number within the long ROH category (>5 Mb).

The results obtained from SNP array data are in concordance with those obtained from genealogical data, which revealed a relatively high effective population size (111.24), low average inbreeding coefficient (0.42%) and a high number of founder animals (179) for the Istrian shorthaired hound. The inbreeding coefficient calculated from genealogical data is largely dependent on the amount of pedigree information available [49]. Therefore, it is important that we were able to confirm a low inbreeding coefficient, also using molecular data. The higher observed heterozygosity compared to expected heterozygosity and a low proportion of inbred animals in the population of Istrian shorthaired hound indicate together with a low proportion of long regions of homozygosity, a low level of recent inbreeding, which could potentially play a detrimental role for the health of the population [51].

### 4.2. Clustering of Breeds

As already proposed by Parker et al. (2017) [10] and Talenti et al. (2018) [9], the dog breeds can be divided into three groups using PCA: western breeds, Mediterranean breeds, and breeds of Asian origin. According to SNP data, the Istrian shorthaired hound clearly belongs to the Mediterranean group of breeds. Several Italian hunting dog breeds are also placed in this group, including Segugio Italiano a Pelo Forte, Segugio Italiano a Pelo Raso, Bracco Italiano, Lagotto Romagnolo, and Spinone Italiano. According to PCA and NeighborNet network, the Istrian shorthaired hound has the closest genetic relationship with both Segugio Italiano breeds (a Pelo Forte and a Pelo Raso). This was confirmed also in the ADMIXTURE analysis, where Bracco Italiano, Lagotto Romagnolo, and Spinone Italiano split off earlier from the cluster, composed of Istrian shorthaired hound and both Segugio Italiano breeds.

In the past, the close relatedness between the Dalmatian dog and the Istrian shorthaired hound, based on analysis of 16 microsatellite loci [5], presence of specific urate metabolic pathway characteristic for the Dalmatian dog and also observed in Istrian shorthaired hound individuals [52], and light color spot pattern similar to the lemon-colored spots of the Dalmatian, was suggested [53]. However, the results of the NeighborNet network show that there is only a very distant relationship between these two breeds. The ADMIXTURE analysis showed that the Dalmatians share the genetic origin with the Dachshund. In contrast to other dog breeds, where breeds that differ in anatomical characteristics and behavior, are close to each other within the clades due to their close geographical origin (e.g., Cane Corso and Lagotto Romagnolo in the Mediterranean clade or Border Collie and Pointer in the UK rural clade) [10], this was not observed for the Istrian shorthaired hound and Dalmatians, although these two breeds originate from geographically very close regions in Croatia.

### 4.3. Phenotypic and Hunting Traits

In the TreeMix analysis, a weak contribution of Beagles was detected to both Segugio breeds. This could be the consequence of some crossing attempts in order to improve their hunting performance. Alternatively, this corresponds to the theory that selection for a certain behavior, even in geographically distant breeds with anatomical differences, can contribute to the development of similar genetic backgrounds [54]. The same analysis also showed some introgression of Beagle to the early ancestors of the Istrian shorthaired hound. The breeding program for Istrian shorthaired hound does not exist, therefore, this introgression might be the consequence of some sporadic crossings, rather than deliberate efforts to improve the breed.

The genomic regions with the highest frequency of ROH (ROH islands) which are potentially under selection [55] were revealed. ROH island on CHA10 spans genes *WIF1*, *LEMD3*, and *MSRB3*. Gene *WIF1* is associated with ear morphology in pigs [32] and dogs [33]. Genes *LRGUK* and *EXOC4* in ROH island on CHA14 are associated with diabetes and fasting glucose in human [34], and could be associated with endurance in this dog breed as it was shown that fasting glucose is significantly correlated with the intensity of the training regimen in professional athletes [56]. Furthermore, two ROH islands (CFA28 and CFA30) including genes *NHLRC2*, *ADRB1*, *CCDC186*, *TDRD1*, *VWA2*, *AFAP1L2*, and *RYR3* were previously identified as genomic regions under strong selection in sport-hunting breeds dog [36]. ROH island on CFA22 includes genes *SETDB2*, *CAB39L*, *CDADC1*, *MLNR*, and *FNDC3A*. Polymorphisms within gene *MLNR* were identified in the study comparing pointing and herding dog breeds [35].

The results of the iHS analysis correspond to the results of Freedman et al. (2016) [57], who reported that signals in the top 100 regions consistent with positive selection in dog genome were frequently centred on candidate genes related to brain function and behavior. The strongest signals in Istrian shorthaired hound were found on chromosomes 15, 14, 4, 31, and 1, and the majority of identified loci were associated with neurological and sensory functions. However, the strongest signal at CFA1 was located in a region where no genes are annotated in the present canine genome assembly; therefore, the interpretation of the biological relevance of this signal is not possible yet. The second strongest signal was identified at CFA14 in the intron of the *DGKB* gene, which is strongly expressed in the olfactory bulb, cortex, striatum, and hippocampus, but not in the thalamus and hypothalamus. *DGKB* is strongly expressed in the rod and cone bipolar cells and horizontal cells of the outer plexiform layer [58]. The intronic region of the *DGKB* gene, identified in this study, might be involved in the expression profile of the *DGKB* gene. In *DGKβ* knockout (KO) mice, cognitive impairment, mania-like behavior, and increased seizure susceptibility were observed [59]. Additionally, in the primary culture of hippocampal neurons from *DGKβ* knockout mice, branching and spine formation have been decreased, however, the phenotype could be rescued in case of *DGKβ* overexpression [59]. Based on these results, *DGKB* could be a good candidate locus for traits that affect changes in synaptic formation and are involved in locomotion and anxiety-related behavioral patterns. The signaling protein *RGS14* is highly enriched in pyramidal neurons and plays a role in the suppression of synaptic plasticity and hippocampal-based learning and memory [60]. In the mouse KO model, RGS14-KO mice exhibited enhancement in spatial learning and object recognition memory. *RGS14* is a member of the G-protein signalling family promoting the conversion of short-term to long-term object recognition memory [61]. In the proximity is also the *LMAN2* (Lectin, Mannose-Binding 2) gene, which encodes a type I transmembrane lectin, and is downregulated in humans with Down syndrome. This complex network of gene functions makes the *RGS14* gene a good candidate for learning associated behavioral traits, which can play an important role in dog learning and training. *Pde10a* (phosphodiesterase 10A) is highly expressed in the brain and is involved in the control of coordinated movement in humans [62]. Homozygous mutation of this gene in mice results in decreased exploratory behavior, hypoactivity, and delay in the acquisition of conditioned avoidance behavior, while hypomorphic allele results in increased social behavior.

## 5. Conclusions

This is the first study estimating the genome-wide population structure of Istrian shorthaired hound. Compared to other dog breeds, the Istrian shorthaired hound has a low inbreeding coefficient and a large number of short runs of homozygosity, confirming the historical information of its ancient origin. The breed displayed a clear genetic differentiation from other dog breeds, placing it into the same clade with Italian hunting dogs, in which development Istrian shorthaired hound most likely has been involved, except Lagotto Romagnolo and Bichonese dog breeds. New positional candidate genes within regions showing evidence of selection associated with cognitive performance and behavior have been identified in the breed. The genomic analysis represents a useful tool for quantifying inbreeding levels and can be implemented in future selection schemes and breeding strategies for the conservation of this old indigenous breed.

The results of the analysis detected several genomic regions, which show genomic signature in the population of Istrian shorthaired hound and represent potential candidates for important behavioral traits in the Istrian shorthaired hound, fundamental for breed-specific behavioral traits that are highly appreciated in this breed. Further analysis of these target regions is necessary to identify possible causal genetic variants specific for this breed.

## Figures and Tables

**Table 1:** Sources of Illumina CanineHD BeadChip datasets included in the study.

Depository	Accession	Number of Samples	Number of Breeds	Reference
GEO	GSE121027	273	21	[9]
GEO	GSE83160	137	22	[8]
GEO	GSE96736	158	47	[10]
Dryad	dryad.v9t5h	5406	163	[11]
Dryad	dryad.1vhhmgqrj	48	1	this study

**Table 2:** Results of pedigree analysis for the reference population of the Istrian shorthaired hound.

Parameter	Reference Population
Number of analyzed animals	520
Number of inbreeds	54
Average inbreeding coefficient	0.42%
Average inbreeding coefficient in inbreeds	4.08%
Average relatedness	1.46%
Inbreeding rate per generation, ΔF	0.45%
Effective population size	111.24
Number of founder animals	179
The effective number of founders	100
The effective number of ancestors	59
Number of maximum generations, mean	2.26
Number of complete generations, mean	1.36
Number of equivalent generations, mean	1.70

**Table 3:** Summary population genetics statistics for 32 investigated dog breeds and Grey wolf.

Breed	Pol. Loci [%]	N	P	H_OBS_ ± SD	H_EXP_ ± SD	π ± SD	Fis ± SD	F_ROH_ ± SD	Ne
Afghan Hound	70.38	10.99	0.82	0.238 ± 0.214	0.237 ± 0.193	0.248 ± 0.202	0.024 ± 0.233	0.295 ± 0.074	32
Beagle	89.87	30.97	0.78	0.277 ± 0.174	0.295 ± 0.174	0.300 ± 0.176	0.062 ± 0.185	0.221 ± 0.083	88
Bergamasco	81.04	9.00	0.79	0.276 ± 0.213	0.282 ± 0.182	0.299 ± 0.193	0.050 ± 0.288	0.229 ± 0.136	28
Bolognese	88.93	17.98	0.77	0.302 ± 0.191	0.304 ± 0.172	0.313 ± 0.177	0.028 ± 0.220	0.162 ± 0.059	55
Bracco Italiano	72.61	8.99	0.81	0.268 ± 0.229	0.253 ± 0.193	0.268 ± 0.204	−0.001 ± 0.238	0.248 ± 0.025	30
Cirneco dell’Etna	83.93	13.87	0.80	0.275 ± 0.202	0.276 ± 0.180	0.286 ± 0.187	0.027 ± 0.238	0.217 ± 0.119	38
Dachshund	93.34	39.96	0.77	0.289 ± 0.166	0.310 ± 0.167	0.314 ± 0.169	0.068 ± 0.167	0.194 ± 0.091	140
Dalmatian Dog	84.90	22.99	0.79	0.265 ± 0.188	0.282 ± 0.181	0.289 ± 0.185	0.064 ± 0.220	0.260 ± 0.053	69
German Shepherd Dog	89.46	47.93	0.81	0.238 ± 0.174	0.255 ± 0.180	0.258 ± 0.182	0.067 ± 0.167	0.326 ± 0.079	86
Grey Wolf	77.20	13.53	0.82	0.200 ± 0.174	0.248 ± 0.186	0.257 ± 0.193	0.158 ± 0.288	0.181 ± 0.170	66
Great Pyrenees	94.38	19.92	0.76	0.261 ± 0.157	0.322 ± 0.160	0.331 ± 0.165	0.177 ± 0.265	0.259 ± 0.129	70
Ibizan Hound	93.54	10.93	0.75	0.307 ± 0.179	0.336 ± 0.166	0.352 ± 0.168	0.111 ± 0.144	0.135 ± 0.107	53
Istrian Shothaired Hound	93.99	47.37	0.77	0.317 ± 0.182	0.311 ± 0.154	0.314 ± 0.162	−0.006 ± 0.298	0.123 ± 0.033	109
Keeshond	78.60	20.98	0.80	0.259 ± 0.204	0.266 ± 0.191	0.272 ± 0.196	0.032 ± 0.218	0.264 ± 0.049	50
Lagotto Romagnolo	86.86	17.94	0.79	0.293 ± 0.198	0.287 ± 0.177	0.296 ± 0.182	0.007 ± 0.205	0.188 ± 0.050	51
Lupino del Gigante	89.17	15.99	0.77	0.302 ± 0.192	0.303 ± 0.171	0.312 ± 0.176	0.028 ± 0.224	0.166 ± 0.089	53
Lupo Italiano	52.06	23.87	0.87	0.185 ± 0.218	0.174 ± 0.198	0.178 ± 0.202	−0.016 ± 0.139	0.451 ± 0.037	20
Mannaras Dog	89.94	11.99	0.76	0.314 ± 0.200	0.315 ± 0.166	0.329 ± 0.173	0.040 ± 0.272	0.129 ± 0.075	42
Maremma	87.19	13.98	0.77	0.310 ± 0.201	0.302 ± 0.173	0.313 ± 0.180	0.009 ± 0.230	0.135 ± 0.056	46
Neapolitan Mastiff	92.05	17.95	0.77	0.271 ± 0.177	0.309 ± 0.167	0.317 ± 0.172	0.125 ± 0.271	0.241 ± 0.104	60
Otterhound	62.81	11.99	0.84	0.231 ± 0.233	0.214 ± 0.199	0.223 ± 0.207	−0.019 ± 0.202	0.335 ± 0.055	24
Pastore d’Oropa	85.43	14.99	0.78	0.339 ± 0.244	0.295 ± 0.179	0.305 ± 0.186	−0.063 ± 0.283	0.115 ± 0.089	45
Pastore della Lessinia e del Lagorai	90.33	10.00	0.76	0.323 ± 0.200	0.319 ± 0.164	0.336 ± 0.172	0.033 ± 0.273	0.108 ± 0.066	32
Pastore della Sila	85.80	13.99	0.78	0.322 ± 0.233	0.290 ± 0.178	0.301 ± 0.185	−0.041 ± 0.261	0.082 ± 0.073	32
Piccolo Levriero Italiano	87.89	29.88	0.79	0.260 ± 0.179	0.284 ± 0.179	0.289 ± 0.182	0.085 ± 0.216	0.266 ± 0.064	72
Portuguese Water Dog	84.85	18.92	0.78	0.269 ± 0.192	0.292 ± 0.182	0.300 ± 0.187	0.087 ± 0.255	0.241 ± 0.073	44
Segugio Italiano a Pelo Forte	92.74	16.00	0.76	0.323 ± 0.186	0.321 ± 0.162	0.331 ± 0.167	0.020 ± 0.226	0.107 ± 0.040	71
Segugio Italiano a Pelo Raso	91.02	16.00	0.77	0.313 ± 0.187	0.313 ± 0.166	0.323 ± 0.172	0.024 ± 0.222	0.131 ± 0.057	64
Spinone Italiano	88.70	17.99	0.77	0.303 ± 0.193	0.303 ± 0.171	0.312 ± 0.176	0.027 ± 0.226	0.157 ± 0.044	60
Standard Schnauzer	87.01	13.93	0.78	0.261 ± 0.186	0.298 ± 0.175	0.309 ± 0.181	0.128 ± 0.290	0.258 ± 0.057	46
Vizsla	86.37	47.96	0.79	0.277 ± 0.189	0.281 ± 0.181	0.284 ± 0.183	0.023 ± 0.162	0.227 ± 0.061	66
Volpino Italiano	88.76	15.96	0.78	0.283 ± 0.191	0.293 ± 0.173	0.302 ± 0.179	0.053 ± 0.241	0.206 ± 0.112	44
Wirehaired Pointing Griffon	87.92	17.99	0.77	0.294 ± 0.191	0.305 ± 0.174	0.314 ± 0.179	0.046 ± 0.237	0.182 ± 0.051	54

Pol. loci [%]—% polymorphic loci; N—the average number of individuals genotyped at each locus; P—average frequency of the most common allele; H_OBS_—average observed heterozygosity; SD—standard deviation; H_EXP_—average expected heterozygosity; π—average nucleotide diversity; Fis—average Wright’s inbreeding coefficient; F_ROH_—average ROH-derived inbreeding coefficient; Ne—effective population size.

**Table 4:** ROH summary: mean number of ROH (N_ROH_), mean ROH length (L_ROH_) and mean genome length covered by ROH (S_ROH_) for Istrian shorthaired hound and four related Italian dog breeds.

	* ROH Category (Mb)	Istrian Shorthaired Hound	Segugio Italiano a Pelo Forte	Segugio Italiano a Pelo Raso	Bracco Italiano	Lagotto Romagnolo
N_ROH_	all	186.42	151.38	157.56	210.89	167.11
	0.5–2.5	159.96	133.13	134.75	152.56	127.17
	2.5–5.0	16.98	8.87	9.81	26.56	17.50
	>5.0	10.59	10.60	13.00	31.78	22.44
L_ROH_ (Mb)	all	1.58	1.69	1.99	2.81	2.70
	0.5–2.5	0.96	0.86	0.89	0.99	0.96
	2.5–5.0	3.41	3.47	3.47	3.61	3.52
	>5.0	8.71	11.30	12.26	10.87	11.88
S_ROH_ (Mb)	all	294.79	255.54	313.59	592.59	450.82
	0.5–2.5	153.58	114.38	120.10	151.37	122.50
	2.5–5.0	57.90	30.79	34.09	95.88	61.60
	>5.0	92.20	119.78	159.40	345.34	266.72
F_ROH_	all	0.12	0.11	0.13	0.25	0.19
	0.5–2.5	0.06	0.05	0.05	0.06	0.05
	2.5–5.0	0.02	0.01	0.01	0.04	0.03
	>5.0	0.04	0.05	0.07	0.14	0.11

* short ROH (0.5 Mb ≤ ROH < 2.5 Mb), medium ROH (2.5 Mb ≤ ROH < 5 Mb), and long (ROH > 5 Mb) ROH per breed.

**Table 5:** ROH islands.

Chr.	Start	End	Protein-Coding Genes	Phenotype	Reference
10	7658201	8019503	*WIF1, LEMD3, MSRB3*	Ear morphology in pigs and dogs (*WIF1*)	[32,33]
14	3249578	3503246	*LRGUK, EXOC4*	Diabetes and fasting glucose in human	[34]
22	2264410	2975487	*SETDB2, CAB39L, CDADC1, MLNR, FNDC3A*	Pointing vs. herding dog breeds (*MLNR*)	[35]
28	24727970	25175110	*NHLRC2, ADRB1, CCDC186, TDRD1, VWA2, AFAP1L2*	Under strong selection in sport-hunting breeds dog (*ADRB1*)	[36]
30	1363393	1523168	*RYR3*	Under strong selection in sport-hunting dog breeds	[36]

**Table 6:** Genomic regions with the highest iHS values.

SNP	Chr.	Position	|iHS|	logP	Gene
BICF2G630421358	15	57356458	4.854	5.917	No annotated genes in this region.
BICF2G630525206	14	29064450	4.564	5.299	Intron 20 of the *DGKB* gene.
BICF2G630167445	4	36002909	4.374	4.915	Upstream gene variant of the gene *RGS14* (3,6 kbp), 5 prime UTR variant of *LMAN2*, downstream variant of the gene *F12* (4.9 kbp).
BICF2S23659818	31	31128590	4.263	4.695	A transcript variant of non-coding RNA gene ENSCAFG00000043029, downstream of the genes *cfa-mir-802* (123.3 kbp) and *SETD4* (22.0 kbp).
BICF2P842400	1	53824762	4.226	4.624	22.8 kbp upstream of the gene *PDE10A.*
BICF2P722838	1	53834552	4.226	4.624	32.6 kbp upstream of the gene *PDE10A.*

iHS—integrated haplotype score.

**Table 7:** Average inbreeding coefficients (F) and inbreeding rate per generation (ΔF) in different dog breeds (data from this study and from the literature).

Breed	Average F (%)	ΔF (%)	Reference
Istrian shorthaired hound	0.42	0.45	this study
Italian pop. of Basset hound	1.10	-	[37]
Alpine Dachsbrake	2.26	-	[39]
Finnishound	3.90	-	[40]
Bracco Italiano	4.10	-	[37]
Bavarian Mountainhound	4.5	0.69	[41]
Finnishound	4.60	-	[42]
Braque Francais type Pyrenees	5.19	-	[43]
German Dachshund	5.23	-	[44]
Hannoveraner Jagdhund	6.80	0.98	[41]
Tiroler Jagdhund	9.50	1.88	[41]
Polish hunting dog	11.51	-	[45]
Norwegian Lundehund	33.00	4.00	[38]

## Supplementary Materials

The following are available online at https://www.mdpi.com/2076-2615/10/11/2013/s1, Figure S1: Plot of ADMIXTURE cross-validation error, Table S1: List of dog breeds with accession numbers used in the study.
