# Supplementary figures and images for: Genomic Characterization of the Istrian Shorthaired Hound
Source: Animals (Basel). 2020 Nov 1;10(11):2013. doi: 10.3390/ani10112013 (PMC7693797; doi:10.3390/ani10112013)

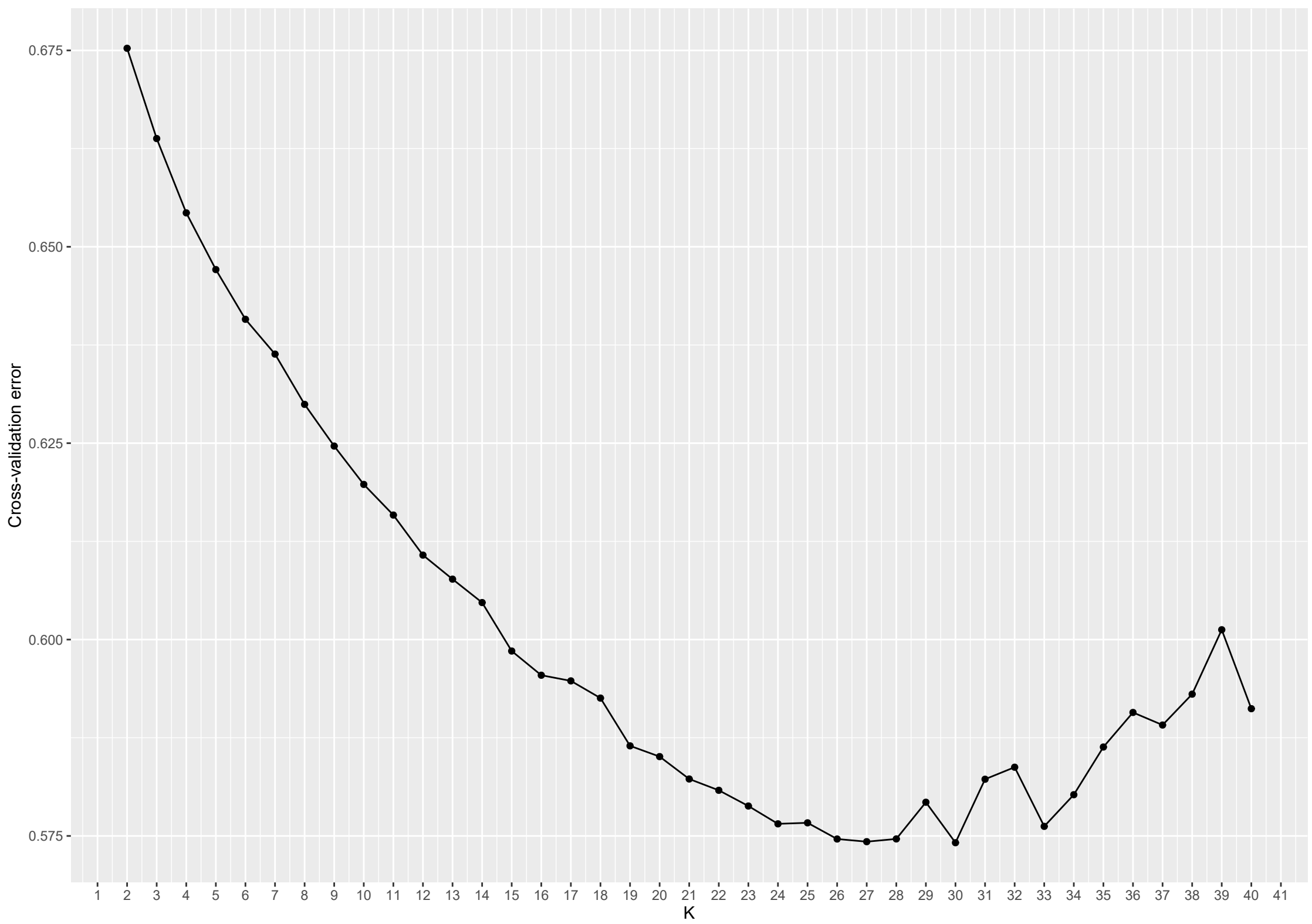

Supplement: Supplementary file 1 [file animals-10-02013-s001.zip › SupplementaryFigure.pdf]
